# Causes and management of persistent septal deviation after septoplasty

**DOI:** 10.1038/s41598-022-23772-y

**Published:** 2022-11-15

**Authors:** Dong-Joo Lee, Hyunju Jo, Ha-Nee Kwon, Ji-Hwan Park, Sung-Dong Kim, Kyu-Sup Cho

**Affiliations:** 1 Department of Otorhinolaryngology and Research Institute for Convergence of Biomedical Science and Technology, Pusan National University Yangsan Hospital, Yangsan, South Korea; 2 Department of Otorhinolaryngology and Biomedical Research Institute, Pusan National University School of Medicine, Pusan National University Hospital, 179 Gudeok-Ro, Seo-Gu, Busan, 602-739 Republic of Korea

**Keywords:** Cartilage, Respiratory signs and symptoms, Bone

## Abstract

Septoplasty is one of the most common otolaryngological surgical procedures. The causes of persistent septal deviation after primary septoplasty vary. The purpose of this study was to identify factors associated with failure of primary septoplasty, operative techniques that correct residual septal deviation, and surgical outcomes. Seventy-four adults who underwent revision septoplasty to treat persistent septal deviations were enrolled. The level of hospital in which primary septoplasty was performed, type of septal deviation, septal portion exhibiting persistent deviation, and techniques used to correct the deviation were evaluated. Outcomes were measured subjectively using a visual analog scale (VAS), and objectively using acoustic rhinometry. The first septoplasties were usually performed in primary and secondary hospitals. C-shaped deviations were more common than S-shaped ones in both the anteroposterior and cephalocaudal dimensions. The most common region of persistent septal deviation was the caudal septum (44.6%), followed by multiple sites (20.3%). The corrective techniques included excision of the remnant deviated portion (70.3%), septal cartilage traction suturing (27.0%), spreader grafting (13.5%), and cross-suturing (6.8%). The VAS score improved significantly 6 months after surgery. The minimal cross-sectional area and nasal cavity volume of the convex side increased significantly after revision septoplasty. Patients who underwent septoplasty in primary and secondary hospitals were more likely to require revision septoplasty. The caudal septum was the most common site of persistent septal deviation. Careful preoperative evaluation of the caudal septal deviation and selection of an appropriate surgical technique may reduce the need for revision septoplasty.

## Introduction

Nasal obstruction is one of the most common symptoms encountered by otolaryngologists in general practice. Although the etiologies vary, a deviated nasal septum is the most common cause of unilateral nasal obstruction^1^; it can make breathing difficult and trigger frequent nosebleeds and repeated sinus infections, as well as headaches, snoring, sleep disturbances, and worsening sleep apnea^2^. Various septoplasty techniques have been used to deal with septal deviations at various sites. Septoplasty can be performed via an endonasal approach or open rhinoplasty.

Septoplasty is one of the most common otolaryngological surgical procedures^3^. However, over 15% of patients who undergo primary septoplasty experience no symptom relief^1,4,5^. Persistent nasal obstruction may be attributable to unrecognized nasal valve issues, inappropriate management of turbinate hypertrophy, exacerbation of allergic rhinitis, and residual or recurrent septal deviation. However, incomplete correction of septal deviation is the main cause of persistent septal deviation after primary septoplasty.

Here, we review the long-term outcomes of revision septoplasty in patients with persistent nasal obstruction after septoplasty. We aimed to identify factors affecting the failure of the primary septoplasty, and evaluated the surgical techniques used to correct residual septal deviation and the postoperative outcomes using subjective symptoms and acoustic rhinometric data.

## Subjects and methods

### Subjects

We retrospectively reviewed the medical records and endoscopic findings of 96 patients who underwent revision septoplasty at Pusan National University Hospital from March 2011 to February 2020. All patients were aged ≥ 18 years and underwent revision septoplasty because of clinically significant residual septal deviations despite prior septoplasty. The exclusion criteria included contemporaneous endoscopic sinus surgery (ESS), nasal valve surgery or nasal polypectomy, a history of facial trauma, a history or clinical evidence of allergic rhinitis, rhinosinusitis, atrophic rhinitis, septal perforation, bleeding or disorder/anticoagulant therapy, pregnancy, and any severe medical or neuropsychiatric disorder. This study protocol was approved by the Institutional Review Board of Pusan National University Hospital (H-2105-007-102) and performed in accordance with relevant guidelines and regulations. Informed consent was waived by the Institutional Review Board of Pusan National University Hospital (H-2105-007-102) due to retrospective nature of study.

### Surgical procedure

All revision septoplasties were endoscopically performed under general anesthesia, using an endonasal approach or external rhinoplasty approach. The latter approach was used only when it was important to maximize visualization and access because of a need for particularly complex septal reconstruction. Various techniques were used to correct the residual septal deviations. Patients with turbinate hypertrophy underwent inferior turbinate out-fracture and volume reduction using a microdebrider. Silicone nasal splints were inserted and removed at 7 days postoperatively. Routine postoperative saline nasal irrigation and debridement were performed.

### Data collection and outcome assessment

We obtained clinical data including age, gender, side of nasal obstruction, level of the hospital where primary septoplasty was performed, time between primary and revision septoplasty, surgical approach, use of combined procedures, techniques used to correct residual deviation, and any complications.

**Figure 1:**
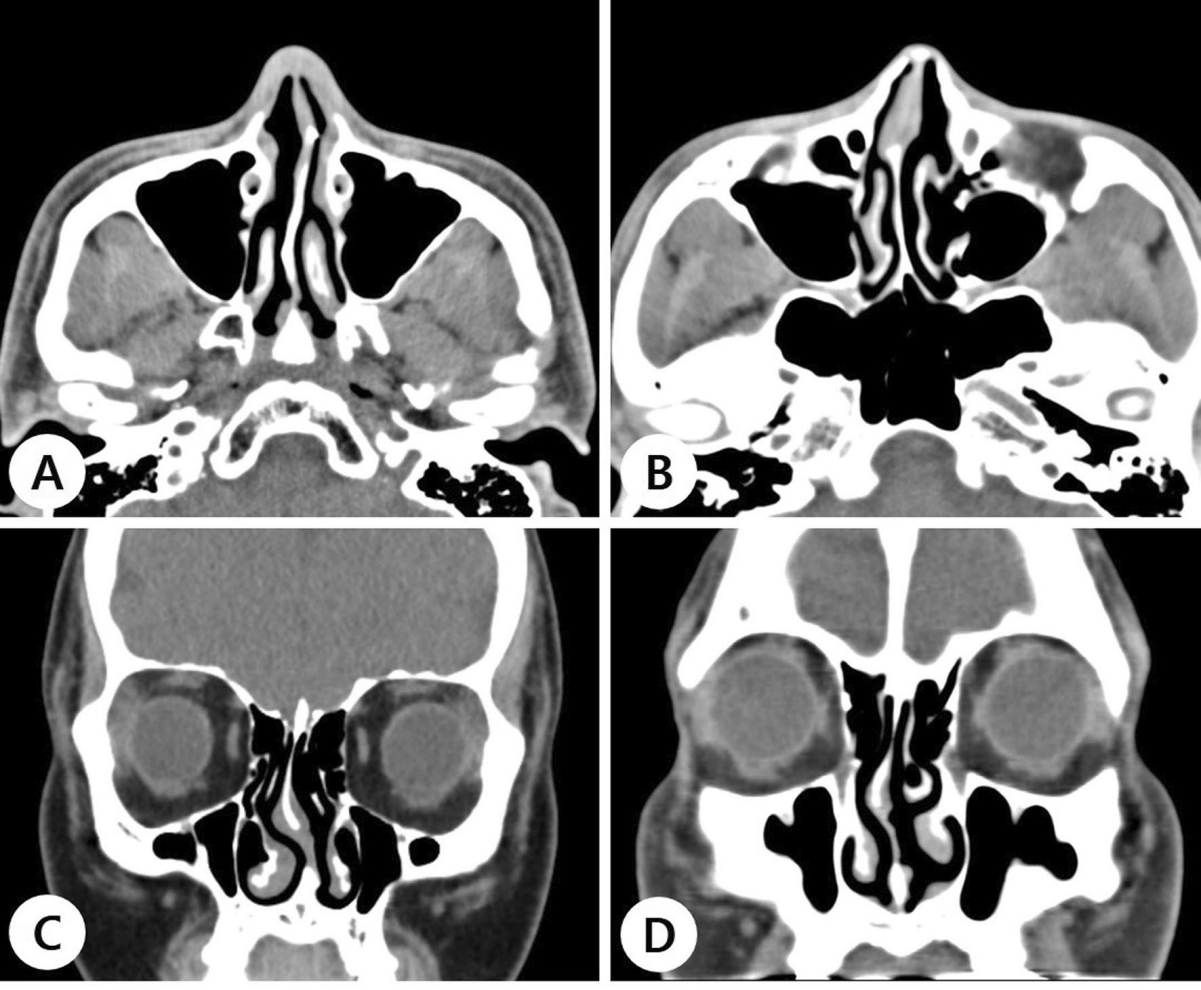
Types of nasal septal deviation. Axial (**A**, **B**) and coronal (**C**, **D**) computed tomography images reveal S- (**A**, **C**) and C-shaped (**B**, **D**) deviations in the anteroposterior and cephalocaudal directions, respectively.

The septal deviation type and region of persistent septal deviation were defined using computed tomography (CT). Both S- and C-shaped deviations were divided into the anteroposterior and cephalocaudal dimensions (Fig. 1).

**Figure 2:**
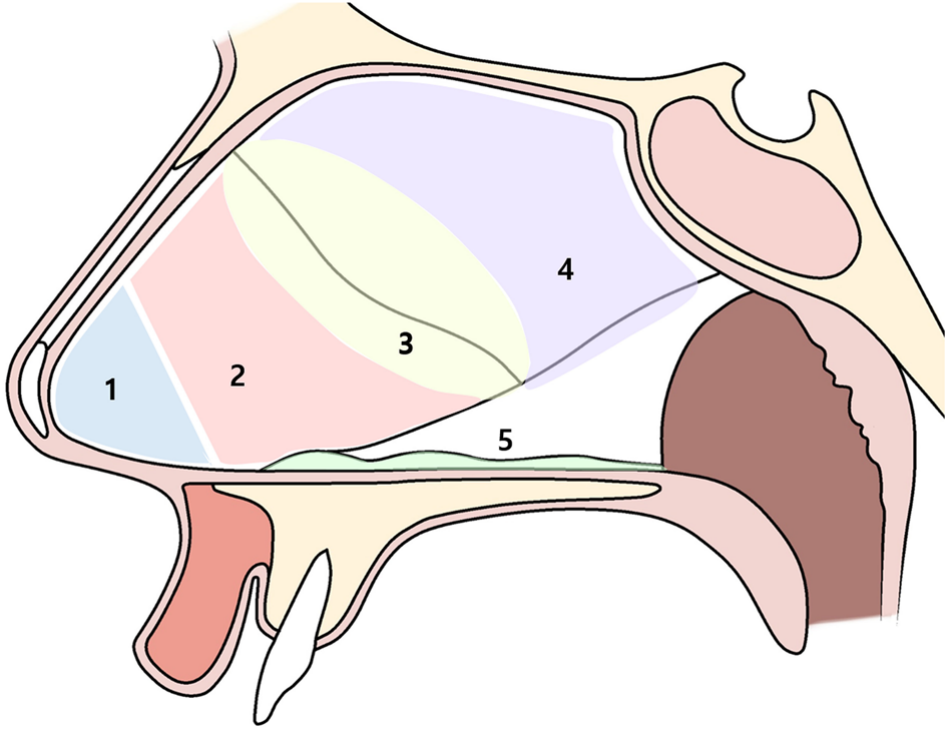
Classification of nasal septal deviations. Persistent septal deviations were seen in the caudal (1), anterior (2), middle (3), posterior (4) septum, and in the maxillary crest (5).

The locations of persistent septal deviation were classified as follows: caudal septum (caudal end of the cartilaginous septum), anterior septum (cartilaginous septum except the caudal septum), middle septum (septum around the cartilage/bone junction), posterior septum (bony septum), maxillary crest, and multiple sites (Fig. 2).

Each patient was evaluated preoperatively and at the 6-month follow-up. Subjective outcomes were measured using a visual analog scale (VAS). VAS scores ranged from 0 to 10 (0, no obstruction; 10, complete obstruction). Objective outcomes were evaluated by measuring the minimal cross-sectional area (MCA) and volume of both nasal cavities using acoustic rhinometry.

### Statistical analysis

Data are presented as mean ± standard deviation. Statistical significance was determined using the paired t-test, performed with SPSS software (ver. 23.0; SPSS Inc., Chicago, IL, USA). A *p*-value ˂ 0.05 was considered to indicate statistical significance.

## Results

Of 96 patients who underwent revision septoplasty, 19 (19.8%) were excluded because they underwent simultaneous ESS. Two patients had experienced prior septal perforations and one was excluded because of a nasal bone fracture; we finally analyzed seventy-four patients. The 66 (89.2%) males and 8 (10.8%) females ranged in age from 18 to 70 years (mean age = 37.5 years). Thirty-nine (52.7%) and thirty-five (47.3%) patients had unilateral and bilateral obstructions, respectively. The first septoplasty had been performed in primary (32/74, 43.2%), secondary (32/74, 43.2%), and tertiary (10/74, 13.6%) hospitals. The interval between primary and revision septoplasty was 11.30 ± 5.82 years (range: 1 ~ 39 years). Sixty-one patients (82.4%) underwent revision endonasal septoplasty and thirteen (17.6%) underwent septorhinoplasty using an external approach. Inferior turbinate out-fracture and turbinoplasty were combined in 45 patients (73.8%) who had undergone septoplasty and 5 who had undergone septorhinoplasty (38.5%). Only one patient developed a postoperative complication (septal perforation) during the 6-month follow-up. The patients’ characteristics are summarized in Table 1.

**Table 1:** Patient characteristics.

Age (years)	37.50 ± 14.76
**Sex**	
Male	66 (89.2)
Female	8 (10.8)
Nasal obstruction	
Unilateral	39 (52.7)
Bilateral	35 (47.3)
**Type of hospital**	
Primary	32 (43.2)
Secondary	32 (43.2)
Tertiary	10 (13.6)
Time between primary and revision surgery (years)	11.30 ± 5.82
**Combined surgery**	
Septoplasty	16 (21.6)
Septoplasty with turbinoplasty	45 (60.8)
Septorhinoplasty	8 (10.8)
Septorhinoplasty with turbinoplasty	5 (6.8)
**Complication**	
No complication	73 (98.6)
Septal perforation	1 (1.4)

Data are expressed as the number (percentage) except age and time (means ± standard deviation).

Anteroposterior C-shaped deviations were apparent in 59 patients (79.7%), and S-shaped deviations in 15 (20.3%). In the cephalocaudal dimensions, 65 patients (87.8%) had C-shaped deviations and 9 (12.2%) had S-shaped deviations. The most common location of persistent septal deviations was the caudal septum (44.6%), followed by multiple sites (20.3%), the anterior septum (14.9%), the middle septum (12.1%), the posterior septum (6.8%), and the maxillary crest (1.3%) (Table 2). The corrective techniques included excision of remnant deviated portions (70.3%), septal cartilage traction suturing (27.0%), spreader grafting (13.5%), and cross-suturing (6.8%) (Table 3).

**Table 2:** Locations of persistent septal deviation.

Location	Number (%)
Caudal septum	33 (44.6)
Multiple sites	15 (20.3)
Anterior septum	11 (14.9)
Mid septum	9 (12.1)
Posterior septum	5 (6.8)
Maxillary crest	1 (1.3)

**Table 3:** Main operative techniques to correct the remnant septal deviation.

Technique	Number (%)*
Excision of remnant deviation (cartilaginous or bony portion)	52 (70.3)
Septal cartilage traction suture technique	20 (27.0)
Spreader graft	10 (13.5)
Crossing suture technique	5 (6.8)

* The numbers are not mutually exclusive.

The symptomatic VAS scores for nasal obstruction decreased significantly from 7.84 ± 1.92 to 2.70 ± 2.62 at 6 months postoperatively (*p* < 0.001). The mean MCA and nasal cavity volume of the convex sides increased significantly from 0.39 ± 0.24 cm^2^ and 5.38 ± 2.20 cm^3^ to 0.66 ± 0.19 cm^2^ and 8.04 ± 3.57 cm^3^ at 6 months post-surgery, respectively (*p* = 0.002 and *p* = 0.025, respectively). However, the mean MCA and nasal cavity volume of the concave side did not differ pre- and post-operatively (Table 4).

**Table 4:** Changes in subjective and objective outcomes after revision septoplasty.

Parameter	Preop	6 Mo	*P* Value
VAS	7.84 ± 1.92	2.70 ± 2.62	< 0.001
**MCA (cm**^**2**^**)**			
Convex	0.39 ± 0.24	0.66 ± 0.19	0.002
Concave	0.98 ± 0.57	1.60 ± 1.99	0.237
**NCV (cm**^**3**^**)**			
Convex	5.38 ± 2.20	8.04 ± 3.57	0.025
Concave	8.51 ± 2.95	10.00 ± 3.74	0.273

Data are expressed as means ± standard deviation. MCA = minimal cross sectional area; Mo = month; NCV = nasal cavity volume.

## Discussion

Septoplasty is typically indicated when patients complain of unilateral or bilateral nasal obstruction attributable to structurally deviated cartilaginous or bony portions of the nasal septum. Although various surgical techniques are used to correct septal deviation, the success rates of primary septoplasty range from 43 to 85%^1,5,7,8^. The most common cause of septoplasty failure is residual or recurrent septal deviation attributable to inadequate correction of the deformity^6^. Revision septoplasty is safe for patients with ongoing nasal obstructions and persistent septal deviations after prior septoplasty. We found that patients who had undergone septoplasty in primary and secondary hospitals were more likely to require revision septoplasty than those initially treated in tertiary hospitals. The higher septoplasty failure rates in primary and secondary hospitals may reflect incomplete correction because of a lack of surgical skill or concerns about cosmetic complications such as tip ptosis and saddle nose deformity.

A few studies have explored the sites of persistent septal deviation after primary septoplasty. Gillman et al.^1^ reported that residual deviation was most common in the dorsal septum. However, Becker et al.^4^ found that multiple sites of deviation was most commonly encountered during revision surgery, followed by caudal deviation. Another study found that the middle septum was the most common site of persistent deviation, followed by the caudal septum^6^. In the present study, C-shaped deviations were more common than S-shaped ones in both the anteroposterior and cephalocaudal dimensions as revealed by CT. The most common regions of persistent septal deviation were the caudal septum, followed by multiple sites and the anterior and middle septum.

Caudal septal deviation is a deviation of the most anterior part of the nasal septum; it may cause severe nasal obstruction and significant cosmetic deformities of the nasal base^9–11^. A deviated caudal septum may change the relationship between the lobule and columella, thereby significantly affecting nasal tip position and symmetry^10^. Correction of such a deviation may be difficult; even a small residual deviation may cause severe nasal obstruction and the intrinsic cartilage-bending memory is hard to break^11^. Furthermore, weakening of the caudal septum and separation thereof from the anterior nasal spine can lead to overcorrection, saddle nose deformity, and tip ptosis^12^. Although various techniques have been used to manage caudal septal deviations, this region, along with high septal deviation, is one of the most difficult to surgically correct.

Surgical approaches to revision septoplasty include endonasal and open rhinoplasty. Many techniques (e.g., the “swinging door” method, septal batten or spreader grafting, cutting and suturing, and extracorporeal septoplasty) have been used to correct residual septal deviations, depending on the characteristics thereof and surgeon preferences^6,13,14^. In previous studies, the open approach was more common than the endonasal approach [4.6]. However, 61 (82.4%) and 13 (17.6%) of our patients underwent endonasal and open approaches, respectively. To correct remnant septal deviation, careful excision of residual deviated cartilaginous or bony portions is typically performed. Septal cartilage traction suturing (Seo et al.)^15^ was performed in 20 cases with a deviated caudal septum, and cross-suturing (Joo et al.)^16^ was performed in 5 cases. Spreader grafts were placed in 10 patients to correct deviations of the anterior septum that reached the dorsal septum; an open approach was employed. Revision septoplasty significantly improved subjective symptoms as measured by the VAS. Objective nasal obstruction improvements were measured by acoustic rhinometry. The MCA and nasal cavity volume of the convex side increased significantly 6 months after surgery. However, the MCA and nasal cavity volume of the concave side also increased, probably reflecting volume reduction of the inferior turbinate.

Although this study had the inherent limitations of a retrospective review, we believe that our prospectively collected, validated outcome measurements support the utility of revision septoplasty for patients with persistent nasal obstructions following primary septoplasty. Furthermore, remodeling may play an important role in shaping the final anatomical contour of the nasal septum due to the long interval between primary and revision surgery. Additional well-designed randomized controlled prospective studies are needed to confirm our findings.

## Conclusion

Patients who underwent septoplasty in primary and secondary hospitals were more likely to require revision septoplasty than those seen in tertiary hospitals, typically because of incomplete correction of the caudal septal deviation. Careful evaluation of the caudal septal deviations and selection of appropriate primary surgery techniques may reduce the need for revision septoplasty.

## Supplementary Information

Supplementary Information.

## Data Availability

The datasets used and/or analysed during the current study are deidentified and included in this published article (and its Supplementary Information files).
